# MicroRNAs As Potential Targets for Abiotic Stress Tolerance in Plants

**DOI:** 10.3389/fpls.2016.00817

**Published:** 2016-06-14

**Authors:** Varsha Shriram, Vinay Kumar, Rachayya M. Devarumath, Tushar S. Khare, Shabir H. Wani

**Affiliations:** ^1^Department of Botany, Prof. Ramkrishna More Arts, Commerce and Science College, Savitribai Phule Pune UniversityPune, India; ^2^Department of Biotechnology, Modern College of Arts, Science and Commerce, Savitribai Phule Pune UniversityPune, India; ^3^Molecular Biology and Genetic Engineering Section, Vasantdada Sugar InstitutePune, India; ^4^Division of Genetics and Plant Breeding, Faculty of Agriculture WADURA, Sher-e-Kashmir University of Agricultural Sciences and TechnologyKashmir, India

**Keywords:** abiotic stress, microRNA, post-transcriptional regulation, stress-responses, transgenics

## Abstract

The microRNAs (miRNAs) are small (20–24 nt) sized, non-coding, single stranded riboregulator RNAs abundant in higher organisms. Recent findings have established that plants assign miRNAs as critical post-transcriptional regulators of gene expression in sequence-specific manner to respond to numerous abiotic stresses they face during their growth cycle. These small RNAs regulate gene expression via translational inhibition. Usually, stress induced miRNAs downregulate their target mRNAs, whereas, their downregulation leads to accumulation and function of positive regulators. In the past decade, investigations were mainly aimed to identify plant miRNAs, responsive to individual or multiple environmental factors, profiling their expression patterns and recognizing their roles in stress responses and tolerance. Altered expressions of miRNAs implicated in plant growth and development have been reported in several plant species subjected to abiotic stress conditions such as drought, salinity, extreme temperatures, nutrient deprivation, and heavy metals. These findings indicate that miRNAs may hold the key as potential targets for genetic manipulations to engineer abiotic stress tolerance in crop plants. This review is aimed to provide recent updates on plant miRNAs, their biogenesis and functions, target prediction and identification, computational tools and databases available for plant miRNAs, and their roles in abiotic stress-responses and adaptive mechanisms in major crop plants. Besides, the recent case studies for overexpressing the selected miRNAs for miRNA-mediated enhanced abiotic stress tolerance of transgenic plants have been discussed.

## Introduction

Plants being sessile organisms, persistently face adverse environmental perturbations termed as abiotic stresses, most important being drought, soil salinity, extreme temperatures, and heavy metals. Abiotic stresses have become a major challenge due to their widespread nature and the devastating impacts on plant growth, yields and the quality of plant produce. However, plants have developed intricate mechanisms for sensing and responding to environmental changes (Wani et al., 2016).

To turn on protective mechanisms, plants trigger a network of genetic regulations including altered expression of large proportion of genes by transcriptional and/or translational regulations (Ku et al., 2015). Plants up-regulate the protective genes while down-regulating the negative regulators. Several protein-coding genes have been recognized in recent years for controlling plant responses to abiotic stresses; however, our knowledge on the regulatory mechanisms involved in this response are still limited and necessitates transformative tools to adapt crops to harsh environments (Zhang and Wang, 2015). These post-transcriptional regulations are pivotal for plants to restore and re-establish their cellular homeostasis during and recovery from stress phases, respectively (Sunkar et al., 2012).

Recent research indicates that plants assign miRNAs as critical post-transcriptional gene-expression regulators to attenuate plant growth and development under stress conditions, though how this is achieved at molecular levels is yet to be understood with greater details. The miRNAs represent a widespread class of small (20–24 nt) endogenous RNAs (Zhang, 2015) and regulate the gene expression via directing mRNA cleavage, translational repression, chromatin remodeling, and/or DNA methylation. Usually, stress-upregulated miRNAs down-regulate their target mRNAs, whereas, their suppression leads to accumulation and function of positive regulators (Chinnusamy et al., 2007). Several studies have confirmed that abiotic stress conditions induce aberrant expressions of miRNAs in plants. High throughput sequencing and computational approaches have been used in recent year for identifying a large number of stress-related miRNAs. These findings indicate that miRNAs might serve as potential targets for genetic manipulations to engineer abiotic stress tolerance in plants. We summarize herein recent updates on plant miRNAs, their biogenesis, target-genes, and their regulatory roles in abiotic stress-responses and adaptive mechanisms deciphered in major crop plants. Besides, the overexpression of selected miRNAs for miRNA-mediated plant stress tolerance has also been discussed. We have also tried to shed light upon recent successes, current challenges and future directions in this field.

## Plant miRNAs: tiny size major roles

Production of abiotic stress tolerant crops necessitates better understanding of gene-regulation mechanisms employed by the plants in response to these environmental cues. Research focused on deciphering post-transcriptional regulations by non-protein coding small RNAs that consists blocking of specific messenger RNAs (mRNAs) or affecting epigenetic modifications at the transcriptional level has gained unprecedented attention. The family members of these small ribonucleotide sequences are represented by RNA species differing from each other on the basis of their size, biogenesis, mode of action, and/or regulatory role (Mittal et al., 2016). MiRNAs are abundant in plants and are attributed for major roles in post-transcriptional regulations through base-pairing with complementary mRNA targets, particularly transcription factors (TFs) (Li and Zhang, 2016). In plants, post transcriptional gene regulation involves miRNAs, generated by Dicer-like 1 (DCL1) from miRNA precursors that are transcribed from miRNA genes (Mangrauthia et al., 2013). Plant miRNAs along with TFs constitutes two major families of gene regulators, and these molecules are suggested to share similar regulatory logistics besides participating in cooperative activities in gene regulatory networks (Lin et al., 2012).

The discovery of miRNA genes in *Caenorhabditis elegans* (Lee et al., 1993) has been followed by a sharp increase in identification of more and more plant miRNA families, making them a research hotspot (Cao et al. 2014; Supplementary Table S1 and Supplementary Figure S1). A steady progress in investigations involving miRNAs has led to a better understanding of transcriptional and post-transcriptional level gene-regulatory mechanisms in plants (Sunkar et al., 2012; Zhang, 2015). These tiny-sized non-coding molecules derived from stem-loop structures are regarded as ubiquitous repressors of gene-expression as they fine-tune the target gene expression and degrade and/or inhibit protein production in higher eukaryotes (Akdogan et al., 2015). It exemplifies the emerging view that miRNAs rival the proteins in regulatory importance (Mishra et al., 2015). Nevertheless, this regulation is highly critical for all biological processes as well as stress-response and acclimatization, thus has a big impact on life processes of plants as indicated by several recent investigations (Zhang and Wang, 2015; Li and Zhang, 2016). MiRNAs control the gene expression via causing epigenetic changes besides controlling targets at post-transcriptional level (Khraiwesh et al., 2010; Wu et al., 2010).

Recent findings affirmed that miRNAs play an array of important roles in plant growth, development and metabolism along with their involvement in abiotic stress and pathogen responses (Yang C. et al., 2013; Xie et al., 2015). MiRNAs have been reported as key regulators of plant root development architecture via targeting AUXIN RESPONSE TRANSCRIPTION FACTOR (ARFs) (Khan et al., 2011). On similar lines, in a recent study, Ripoll et al. (2015) observed miRNA-regulated fruit growth in *Arabidopsis*. Besides, plant miRNAs are also known to target genes involved in processes such as sulfur assimilation and ubiquitin-dependent protein degradation (Bonnet et al., 2004). Since vegetative parts are important in crop plants for harvesting reasons, therefore control of plant apical dominance and vegetative growth is significant. There is increasing evidence that miRNAs play crucial role in leaf-development, apical dominance and biomass production, and targeting specific miRNAs is proving a novel and potent strategy for improving plant growth, biomass and crop yield (Zhang and Wang, 2015).

Computational predictions coupled with experimental approaches have led to the conclusion that many TFs including MYB are indeed miRNA targets. Deep sequencing has confirmed the critical roles miRNAs play in cotton ovule and/or fiber development (Xie et al., 2015). They regulate key genes involved in the floral induction and flower formation processes such as transition phases from juvenile to adult, initiation of floral-competence and flower development (Hong and Jackson, 2015). Fruit and seed development held a distinct place in plant propagation and harvesting for defining crop yields, therefore, the roles played by the miRNAs in development of fruits and seeds is of great interest. Itaya et al. (2007) identified a large number of species-specific miRNAs from tomato (*Solanum lycopersicon*) and hypothesized their significant roles in fruit development. Further, tissue-specific expression of numerous miRNAs was reported by Moxon et al. (2008) and the authors proposed miRNA-regulated fleshy fruit development and ripening in *S. lycopersicon*. A number of miRNAs have been identified for targeting metabolic pathways of grain-filing and nutrient-biosynthesis including carbohydrate and protein metabolism, cellular transport and signal transduction besides phytohormone signaling (Meng et al., 2013; Peng et al., 2013; Zhang and Wang, 2015). MiRNAs have been attributed for vital functions in somatic embryogenesis in cotton (*Gossypium hirsutum*) as revealed by the high-throughput and degradome sequencing (Yang X. et al., 2013). Interestingly, deciphering the regulatory roles of miRNAs is not confined only to primary metabolites. Recently, it was revealed that certain miRNAs target transcripts related to secondary metabolism as well (Boke et al., 2015; Bulgakov and Avramenko, 2015) and of these *miR156, miR163, miR393*, and *miR828* have been ascribed as interesting tools for regulating secondary metabolism in *Arabidopsis*.

## Plant miRNAs: their biogenesis, targets and mode of action

Much research, ever since the first report on plant miRNAs in 2002 (Reinhart et al., 2002) has resulted in a considerable gain in our understanding of their origin. Owing to the ubiquitousness and diversity of plant miRNAs, it is apparent that most, if not all, biological processes in plants at some point involve the action of one or more miRNAs (Voinnet, 2009). Plant miRNAs share many similarities with their animal counterparts as revealed by the recent genetic and biochemical studies focused on deciphering their origin, biogenesis and modes of action. These small but fundamental molecules are synthesized from primary (pri)-miRNA transcripts, which are in turn transcribed usually by RNA pol II or pol III from the nuclear-encoded miRNA (MIR) genes. The pri-miRNA transcripts form double stranded stem-loop (imperfectly paired hairpin) structured precursor RNAs (pre-miRNA). In plants, apparently RNA-binding protein DAWDLE (DDL) stabilizes the pri-miRNAs for their conversion to stem-loop pre-miRNAs in D-bodies. This reaction requires a concentrated action and physical interaction of SERRATE (SE, a C2H2-zinc finger protein), a ds-RNA binding protein HYPONASTIC LEAVES1 (HYL1), DCL-1 and nuclear cap-binding complex (CBC) (Fang and Spector, 2007; Gregory et al., 2008; Laubinger et al., 2008; Voinnet, 2009). The pre-miRNA hairpin precursor then gets converted into 20–22 nt miRNA::miRNA^*^ duplex, where miRNA acts as guide-strand whereas miRNA^*^ as degraded strand by the actions of DCL1, HYL1, and SE. This duplex then undergoes methylation at 3′ terminus by HUA ENHANCER1 (HEN1) to protect them against degradation by small RNA degrading exonuclease, SDN. The pre-miRNAs or the mature miRNAs are then exported from nucleus to the cytoplasm by the plant exportin protein HASTY (Chaves et al., 2015). One strand of the duplex in cytoplasm gets unwound into a single stranded mature-miRNA which then gets incorporated into an ARGONAUTE protein, and directs RNA induced silencing complex (RISC) binding to cognate target transcripts through sequence complementarities (Voinnet, 2009; Khraiwesh et al., 2010, 2012).

The successful identification and binding of miRNA-AGO complex with specific mRNA-targets negatively regulate their expression via mRNA cleavage, decay, and/or post-transcriptional repression (Jones-Rhoades et al., 2006). Though there is clear evidence that the level as well as positions of complementarities between the mRNA targets and miRNAs defines the target selectivity, however, there is possibility of involvement of other factors as well (Wang et al., 2015). Since all the miRNAs regulate plant growth and development as well as stress-responses via targeting individual protein-coding genes, therefore identification of these mRNA targets is the first and most important step in elucidating the functions of miRNAs (Zhang and Wang, 2015). Once incorporated in RISC as discussed above, usually the mature miRNA regulate the expression of target genes via three mechanisms. It induces mRNA-slicing of specific target-gene and it is a main pathway of miRNA action in plants (He et al., 2014), whereas second pathway include the inhibition of translation of functional proteins through combining target gene's mRNA and third one incorporates silencing of multi-genes at transcriptional level via chromosomal isochromatin (He et al., 2014). Owing to the fact that plant miRNAs represents the non-coding regions of the genes, they act as an independent transcription unit. TFs are their major targets and have therefore critical roles in hormonal signal transduction, cellular metabolism, organ differentiation, floral patterning and reproduction, as well as in nutrient homeostasis and plant responses to stresses (Sun, 2012; Hong and Jackson, 2015; Tripathi et al., 2015).

## Identification and target prediction of plant miRNAs: computational tools and resources

To identify miRNAs in plants, both experimental as well as computational approaches have been used so far. Identification of miRNAs in plants started in early 2000s with direct-cloning approaches (Llave et al., 2002; Reinhart et al., 2002). Though, it was not easy to identify large number of miRNAs, owing to their multiple occurrences, small size and methylation status. But advancement in cloning methodologies and computational algorithms resulted in a rapid increase in number of miRNAs identified in plants in last few years (Tripathi et al., 2015). In recent past, the advent of high throughput/deep sequencing has really helped in an exponential growth in the number of plant miRNAs, not only identified but also functionally annotated (Jagadeeswaran et al., 2010; Rosewick et al., 2013). This has resulted into establishment of biological databases to act as archives of miRNA-sequences and annotation such as miRBase (Tripathi et al., 2015).

Several computational tools and databases have been developed in recent times to identify and predict the targets of miRNAs using traditional nucleotide databases as well as datasets generated through deep-sequencing approaches, a detailed list is provided in Table 1.

**Table 1:** **A summarized list of major tools available for plant miRNAs, their target identification/prediction and repositories**.

**Name of the database/resource/repository/tool**	**Description**	**Web link**	**References**
TAPIR	Target prediction for Plant miRs	http://bioinformatics.psb.ugent.be/webtools/tapir/	Bonnet et al., 2010
miRTarBase	The experimentally validated miR-target interactions database	http://mirtarbase.mbc.nctu.edu.tw/index.php	Hsu et al., 2011
PMRD	Plant miRNA Database	http://bioinformatics.cau.edu.cn/PMRD/	Zhang et al., 2010
miRanalyzer	miR detection and analysis tool for next-generation sequencing experiments	http://bioinfo5.ugr.es/miRanalyzer/miRanalyzer.php	Hackenberg et al., 2011
PmiRKB	Plant miR Knowledge Base. Four major functional modules, SNPs, Pri-miRs, MiR-Tar and Self-reg, are provided	http://bis.zju.edu.cn/pmirkb/	Meng et al., 2011
miRDeep-P	A computational tool for analyzing the miRtranscriptome in plants	http://faculty.virginia.edu/lilab/miRDP/	Yang and Li, 2011
C-mii	A tool for plant miR and target identification	http://www.biotec.or.th/isl/c-mii	Numnark et al., 2012
Semirna	Searching for plant miRNAs using target sequences	http://www.bioinfocabd.upo.es/semirna/	Muñoz-Mérida et al., 2012
mirTool	A comprehensive web server providing detailed annotation information for known miRs and predicting novel miRs that have not been characterized before	http://centre.bioinformatics.zj.cn/mirtools/	Wu et al., 2013
PASmiR	A literature-curated database for miR molecular regulation in plant response to abiotic stress		Zhang et al., 2013
miRBase	Searchable database of published miR sequences and annotation	http://www.mirbase.org	Kozomara and Griffiths-Jones, 2014
miRPlant	An Integrated Tool for Identification of Plant MiR from RNA Sequencing Data	http://www.australianprostatecentre.org/research/software/mirplant	An et al. (2014)
MTide	An integrated tool for the identification of miR-target interaction in plants	http://bis.zju.edu.cn/MTide/	Zhang et al., 2014b
PNRD	It is an updated version of PMRD	http://structuralbiology.cau.edu.cn/PNRD/index.php	Yi et al., 2015
PlantMirnaT	A miRNA-mRNA integrated analysis system	https://sites.google.com/site/biohealthinformaticslab/resources	Rhee et al., 2015
miRA	Plant miRNA identification tool especially for organisms without existing miRNA annotation. It is also useful for identifying species-specific miRNAs	https://github.com/mhuttner/miRA	Evers et al., 2015
miPEPs	MiRNAs Encode Peptides is a tool for functional analysis of plant miRNA family members		Couzigou et al., 2015
sRNAtoolbox	A set of tools for expression profiling and analysis of sRNA bench results	http://bioinfo5.ugr.es/srnatoolbox	Rueda et al., 2015
miRge	A fast multiplexed method of processing sRNA-sequence data to determine miRNA entropy and identify differential production of miRNA isomiRs	http://atlas.pathology.jhu.edu/baras/miRge.html.	Baras et al., 2015
BioVLAB-MMIA-NGS	MiRNA and mRNA integrated analysis using high-throughput sequencing data coupled with bioinformatics tools.	http://epigenomics.snu.ac.kr/biovlab_mmia_ngs/	Chae et al., 2015
DMD	A dietary miRNA database from 15 dietary plant and animal species	http://sbbi.unl.edu/dmd/	Chiang et al., 2015
WMP	Database for abiotic stress responsive miRNAs in wheat	http://wheat.bioinfo.uqam.ca	Remita et al., 2015
miTRATA	A tool for miRNA truncation and tailing analysis	https://wasabi.dbi.udel.edu/~apps/ta/	Patel et al., 2016
MFSN	A tool for prediction of plant miRNA functions based on functional similarity network (MFSN) through application of transductive multi-label classification (TRAM) to the MFSN		Meng et al., 2016
PlanTE-MIR	Database for transposable element-related plant microRNAs	http://bioinfo-tool.cp.utfpr.edu.br/plantemirdb/	R Lorenzetti et al., 2016
P-SAMS	A Plant Small RNA Maker Site (P-SAMS) is a web tool for artificial miRNAs and synthetic trans-acting small interfering RNAs	http://p-sams.carringtonlab.org	Fahlgren et al., 2016

The miRBase (http://www.mirbase.org/) represents a comprehensive database with searchable online repository of published miRNA sequences and associated annotation. This database was started in 2002 with its first release included just 5 miRNAs from one plant species (*Arabidopsis thaliana*), whereas its latest version (Release 21, June 2014) contains 28,645 entries representing hairpin precursor miRNAs, expressing 35,828 mature miRNA products in 223 species including 73 plant species with 7057 miRNA loci between them (Kozomara and Griffiths-Jones, 2014).

miRNEST represents a wide-ranging database of animal, plant, and virus miRNAs (Szczesniak et al., 2011, http://mirnest.amu.edu.pl). It provides miRNA data on the basis of computational predictions and high-throughput sequencing. It's current (miRNEST 2.0) version contains miRNAs from 22 viruses and >270 plant species. Interestingly, this database also includes degradome data of 2041 entries (as on March 16, 2016).

Plant miRNA database (PMRD, http://bioinformatics.cau.edu.cn/PMRD/) provides information about plant-miRNA sequences as well as their targets (Zhang et al., 2010). It contains the sequence information, secondary structure, target genes, expression profiles and a genome browser. PMRD contains more than 8400 miRNA entries from >120 model plants as well as major crops including *Arabidopsis thaliana*, rice (*Oryza sativa*), wheat (*Triticum aestivum*), soybean (*Glyciene max*), and maize (*Zea mays*), besides providing predicted target-genes and interaction-site in the database. An updated version of PMRD has been launched as PNRD (Plant Non-coding RNA Database) with greater number of entries and plant species as well as broad spectrum RNA types (Yi et al., 2015). There are total 25,739 entries of non-coding RNA (ncRNA) including lncRNAs, tRNA, rRNA, tasiRNA, snRNA, and snoRNA from around 150 plant species, especially food crops. It offers various search and analysis tools for the user such as ncRNA keyword search, for example ID search, target search, and toolkits for predicting online novel miRNAs and for calculating coding potential along with the availability of BLAST tools (http://structuralbiology.cau.edu.cn/PNRD/index.php).

PMTED (Plant miRNA Target Expression Database, http://pmted.agrinome.org/) is a plant-specific miRNA database, useful to study miRNA functions by inferring their target gene expression profiles among the large amount of existing microarray data. It contains tools to search miRNA targets, retrieve expression data and the user can find out differentially expressed genes (Sun et al., 2013).

The TAPIR (target prediction for plant miRNAs) webserver (http://bioinformatics.psb.ugent.be/webtools/tapir/) predicts targets for plant miRNAs, with two available modes, the first “Fast” mode using the FASTA search engine while the second “Precise” mode using the RNA-hybrid search engine (Bonnet et al., 2010).

miRPlant is an integrated tool for identification of plant miRNA from RNA sequencing data (An et al., 2014, http://www.australianprostatecentre.org/research/software/mirplant). miRPlant works on the strategies specifically developed to identify hairpin excision regions and hairpin structure filtering for plants. Interestingly, it does not need third party tools, rather, it uses a graphic user interface for input and output of the data, and the display of recognized miRNA with RNAseq reads is done with the help of a hairpin diagram.

PlantMirnaT developed by Rhee et al. (2015) is a miRNA and mRNA integrated analysis system via utilizing the sequencing data effectively. Its major features include a short read mapping tool, and an algorithm which takes into consideration the miRNA expression and distribution in target mRNAs (Rhee et al., 2015; https://sites.google.com/site/biohealthinformaticslab/resources).

miTRATA (miRNA-Truncation and Tailing Analysis) is a web-tool for truncation and tailing analysis of miRNA, and utilizes the miRBase (var. 21) and useful to analyze 3′ modifications of miRNAs (Patel et al. 2016). In recent years, thorough miRNA biogenesis has revealed more complex features and secondary structures of their precursors. Consequently, Evers et al. (2015) proposed a freely available public access tool “miRA” (https://github.com/mhuttner/miRA) for identification of plant-miRNA precursors, which is adaptable to heterogeneous and complex precursor populations. Interestingly, Meng et al. (2016) developed a tool to predict the functions of plant miRNAs on the basis of their functional similarity network through application of transductive multi-label classification. Transposable elements (TEs) have been recognized for their prominent roles in determining non-coding regions including miRNAs of the genomes (Gim et al., 2014). In order to develop a plant TE related miRNA database (PlanTE-MIR DB) was proposed very recently by R Lorenzetti et al. (2016). The authors identified more than 150 miRNAs overlapping TEs in 10 plant genomes. This pubic database is hosted at http://bioinfo-tool.cp.utfpr.edu.br/plantemirdb/.

It is noteworthy to mention here that in spite of numerous computational tools to identify plant-miRNAs and their target prediction, there is a lack of curated databases of stress related miRNAs. Zhang et al. (2013) advocated for a need to construct a cohesive database system for data deposit and further applications of plant abiotic stress related miRNAs and accordingly developed PASmiR, which allows the users to retrieve miRNA-stress regulatory entries by keyword-search such as plant species and type of stress (Zhang et al., 2013). Similarly, Remita et al. (2015) proposed a web-based server/database “WMP” dedicated to wheat miRNAs, particularly stress-responsive miRNAs and is available at: http://wheat.bioinfo.uqam.ca.

## miRNAs involved in plant abiotic-stress responses

Through adaptive evolution processes, plants have developed incredible abilities to respond and adapt to challenging external conditions. The cellular and molecular responses of plants to these abiotic stresses are intricate and received great attention of plant breeders and biotechnologists in recent past to decipher them. Plants deploy multitude abiotic stress responsive mechanisms involving regulation of expression of stress-responsive genes to sense and respond to external stimuli. Investigations in recent times have shown that different abiotic stress conditions induce aberrant expression of thousands of protein-coding genes in various plant species (Kumar et al., 2009; Zeller et al., 2009; Turan and Tripathy, 2013; Khare et al., 2015). Many of these stress-induced, often individual, genes for example proline biosynthetic pathway gene P5CS in rice (Kumar et al., 2010), methionine sulfoxide reductase gene (MSRB7) in *Arabidopsis* (Lee et al., 2014) and transcription factor TaERF3 in wheat (Rong et al., 2014) were overexpressed to produce respective transgenic plants to confer enhanced tolerance against singular abiotic stress. However, the regulatory mechanisms of these protein-coding genes are largely unknown (Sun, 2012), in this regard, the miRNAs may prove extremely important in deciphering these gene-regulatory mechanisms and the stress-responses. Though, large numbers of miRNA-families are conserved in flowering plants, however, recent updates indicated that miRNAs may be species-, physiological stage or event-, organ or tissue-, and stress-specific (Valdes-Lopez et al., 2010; Sun, 2012).

Several miRNAs have been recognized as abiotic stress-regulated in important crops and/or model plants under soil salinity (Gao et al., 2011), nutrient deficiency (Liang et al., 2015), UV-B radiation (Casadevall et al., 2013), heat (Goswami et al., 2014), and metal stress (Yang and Chen, 2013; Gupta et al., 2014). However, this stress-regulated miRNA expression does not essentially confirm that the miRNA is involved in plant's adaptation to stress conditions. Zhang et al. (2013) observed differential expression of as many as 1062 miRNAs in 41 plant species under 35 different types of abiotic stresses. Table 2 summarizes recent data on the altered miRNA expression in major crop plants in response to abiotic stresses.

**Table 2:** **Differential expression patterns of miRNAs reported under different abiotic stresses (S- salinity; D- drought; HT- high temperature; C- cold; Cd- cadmium; As- arsenic) in major crops- *Oryza sativa, Triticum aestivum, Saccharum* sp., *Glycine max*, and *Hordeum vulgare***.

**miRNA**	**Crop**	**Response**						**Validated/Putative Target**
		**S**	**D**	**HT**	**C**	**Cd**	**As**	
*miR156*	*O. sativa*							SPL TFs
	*T. aestivum*							SPL
	*Saccharum* sp.							SPL 5
	*G. max*							SPL
*miR159*	*O. sativa*							MYB family TFs
	*T. aestivum*							MYB domain protein
	*Saccharum* sp.							GA-Myb
*miR160*	*O. sativa*							ARF
	*T. aestivum*							ARF
	*H. vulgare*							ARF 13, ARF 17
*miR162a*	*O. sativa*							DCL1
*miR164*	*T. aestivum*							NAC domain containing protein
*miR166*	*O. sativa*							START domain containing protein, HD-Zip TFs
	*T. aestivum*							HD-Zip protein
	*Saccharum* sp.							Class III HD-Zip protein 4
	*H. vulgare*							HD-Zip TFs
*miR167*	*O. sativa*							ARF
	*T. aestivum*							ARF
	*Saccharum* sp.							ARF 17
	*G. max*							ARF
	*H. vulgare*							ARF 8
*miR168*	*O. sativa*							AGO
	*T. aestivum*							AGO
	*Saccharum* sp.							AGO 1
	*H. vulgare*							AGO 1
*miR169*	*T. aestivum*							CBF-B/NF-YA family protein
	*Saccharum* sp.							HAP12-CCAAT-box TF complex
	*O. sativa*							NF-YA or HAP2 TFs
*miR170*	*O. sativa*							Scarecrow-like TF, ACP1
*miR171*	*O. sativa*							Scarecrow-like TF
	*T. aestivum*							Scarecrow-like TF
	*Saccharum* sp.							SCL1
	*G. max*							Ubiquitin carrier protein, Fe-SOD 1
	*H. vulgare*							Protein FAN
*miR172*	*O. sativa*							APETALA2, bZIP TF family protein
	*T. aestivum*							APETALA2-like
	*Saccharum* sp.							APETALA2-like
*miR319*	*O. sativa*							TCP family TF21
*miR390*	*O. sativa*							LRR-RLK
*miR393*	*O. sativa*							TIR1, AFB2, AFB3, F-box domain, LRR containing protein/MYB family TF
*miR394*	*O. sativa*							F-box domain containing protein
*miR395*	*T. aestivum*							ATP sulfurylase
	*G. max*							ATP sulfurylase, Low affinity sulfate transporter
*miR396*	*O. sativa*							GRF TFs, rhodenase-like proteins, kinesin-like protein B
	*T. aestivum*							GRF 3
	*Saccharum* sp.							GRF1
	*G. max*							Cytochrome P450 monooxygenase, NADH-ubiquinone oxidoreductase chain 4
*miR397*	*Saccharum* sp.							LAC-10 precursor
	*O. sativa*							LACs
*miR398*	*Saccharum* sp.							Selenium binding protein
*miR408*	*O. sativa*							SPX, BCP
*miR444*	*O. sativa*							MADS-box TFs
	*H. vulgare*							MIKC-type MADS-box TFs
*miR474*	*O. sativa*							PPR, protein kinase, kinesin, leucine-rich repeat
*miR482*	*T. aestivum*							Transmembrane proton gradient regulation
*miR528*	*O. sativa*							IAR1, CBP/Plastocyanin-like domain containing protein
	*O. sativa*							OsDCL1
	*Saccharum* sp.							Putative LAC
*miR529*	*O. sativa*							SBP-box gene family
*miR530-3p*	*O. sativa*							Hairpin-induced protein 1 domain containing protein
*miR809*	*O. sativa*							Glutaredoxin 2, putative, PPR repeat containing protein
*miR827*	*T. aestivum*							NLA
*miR1318*	*O. sativa*							Calcium binding proteins or Calcium ATPases
*miR1428*	*O. sativa*							Cytochrome c
*miR1432*	*O. sativa*							Calcium binding proteins or Calcium ATPases
*miR1884*	*O. sativa*							AAA ATPase
*miR2871*	*O. sativa*							GT family protein
*miR5049*	*T. aestivum*							Wpk4 protein kinase
*miR5175*	*H. vulgare*							ACC-like oxidase

*In the table differential expression pattern of miRNAs under different abiotic stress environments (salt, drought, high temperature, cold, cadmium, and arsenic) was marked as induced (green box) or repressed (red box) expression. References- Oryza sativa: Ding et al. (2011), Barrera-Figueroa et al. (2012), Zhou et al. (2010), Xia et al. (2012), Liu and Zhang (2012); Triticum aestivum: Xin et al. (2010), Pandey et al. (2014), Akdogan et al. (2015); Saccharam: Bottino et al. (2013), Gentile et al. (2015); Glycine max: Li et al. (2011); Hordium vulgare: Deng et al. (2015), Hackenberg et al. (2015), Kruszka et al. (2014)*.

### Drought and salinity stress

Owing to the widespread challenges these two stress-factors put on plant growth and yield, drought, and salinity stresses have received maximum attention of plant scientists to study their deleterious effects and decrypting the responsive-mechanisms adopted by the plants. Over the past two decades, researchers investigated and identified number of genes to improve stress tolerance of transgenics overexpressing those genes. However, on one hand the resultant transgenic plants showed inadequate improvement largely because the complex genetic interactions and on the other hand their regulation underlying plant stress tolerance are not completely understood (Sunkar et al., 2012). Non-coding RNAs including miRNAs however are turning to be a potent platform to understand and decode stress-responsive transcriptional and post-transcription gene regulations in plants.

A number of drought stress-responsive miRNAs were identified via screening of small RNAs library isolated from *Arabidopsis* (Liu et al., 2008), rice (Zhou et al., 2010), and sugarcane (Gentile et al., 2015) and the count is ever increasing. Liu et al. (2008) evidenced a total of 14 stress-inducible miRNAs in *Arabidopsis*, out of them 10 were NaCl-regulated, 4 drought-regulated and 10 were cold (4°C)-regulated, and *miR168, miR171*, and *miR396* (with TFs as predicted targets) responded to all of the stresses.

In rice, Zhao et al. (2007) reported that *miR169g* and *miR393* are strongly upregulated and transiently induced, respectively by drought. In another study, Zhou et al. (2010) used microarray approach for identify and analyze miRNAs in drought stressed rice at developmental stages ranging from tillering to inflorescence formation. Overall, drought conditions induced differential expressions of 30 miRNAs, out of which 19 were newly reported drought-regulated miRNAs from *Arabidopsis*. Sixteen miRNAs (*miR156, miR159, miR168, miR170, miR171, miR172, miR319, miR396, miR397, miR408, miR529, miR896, miR1030, miR1035, miR1050, miR1088*, and *miR1126*) were downregulated by drought stress while it induced the overexpression of 14 miRNAs (*miR159, miR169, miR171, miR319, miR395, miR474, miR845, miR851, miR854, miR896, miR901, miR903, miR1026*, and *miR1125*). Notably, *miR171, miR319*, and *miR896* were identified in both (overexpressed and repressed) groups (Zhou et al., 2010). Predicted targets of the differentially regulated miRNAs were largely TFs (Zhou et al., 2010). Similarly, drought stress induced expression was observed in 438 miRNAs against 205 under controlled conditions in *Triticum turgidum* leaf and root tissues, and 13 miRNAs were differentially regulated by drought conditions (Kantar et al., 2010). Aberrant miRNA expression was observed in soybean cultivars subjected to drought conditions, with upregulation of *miR166*-5p, *miR169*-3p, *miR1513c, miR397ab*, and *miR*-*seq13* in sensitive cultivars but downregulation in their tolerant counterparts (Kulcheski et al., 2011).

Recently in sugarcane, the expression pattern of miRNA was observed to be dependent on the species, type of stress, tissue (seedlings, leaves, spikelets, and root) growth condition (field, greenhouse, hydroponic culture system). *MiR396* and *miR171* were differentially expressed in the most of the cases (Gentile et al., 2015). Interestingly, overexpression of *osa-miR393* resulted into enhanced salt tolerance, suggesting their regulatory role in salinity tolerance in *Arabidopsis* (Gao et al., 2011). In similar vein, Sun et al. (2015) identified 49 known and 22 novel salt stress-responsive miRNAs in radish (*Raphanus sativus*) and interestingly the target prediction analysis revealed the implication of the target genes in signaling, regulating ion-homeostasis besides modulating the decreased plant growth under salt stress.

### Extreme temperatures

Plants often face extreme temperatures due to geographical conditions and seasonal variations, which negatively affects the plant growth and productivity and plants adjust their gene expression patterns at post-transcriptional levels in response to these inconstant external temperatures. Numerous chilling or heat responsive miRNAs have been detected in plant species (Cao et al., 2014). Wheat seedlings of heat tolerant and susceptible cultivars when exposed to heat stress (40°C) exhibited differential miRNA gene expression with increased expression in many of them, where expression was suppressed in few under heat stress (Xin et al., 2011). Eighteen cold-responsive miRNAs were identified by Lv et al. (2010) in rice with most of them being downregulated by the cold stress (4°C) and the authors hypothesized the miRNAs as ubiquitous regulators in rice. On the other hand, cold stress brought a sharp increase in the expression of *miR812q* in rice plants at the beginning of reproductive phase (Jeong et al., 2011). These observations hold merit, since *miR812q* originates from a sequence-diverged region, has unique sequence, and unlike other family members, is cold-responsive (Jeong et al., 2011; Jeong and Green, 2013). Cold stress induced *miR812q* targets CIPK10 and downregulates the later, interestingly, CIPK transcripts are known abiotic stress tolerance mediators in calcium dependent CBL-CIPK signaling pathway (Jeong and Green, 2013).

Such differential expression may be due to species-specific role of miRNAs in cold response (Jeong and Green, 2013). In a recent study by Zhang et al. (2014a), high-throughput sequencing analyses revealed cold stress-induced upregulation of 31 and downregulation of 43 miRNAs in tea (*Camellia sinensis*) plants and authors identified a large number of target genes using degradome sequencing. Functional analysis of miRNA targets reaffirmed their critical roles in stress response. Similarly, Cao et al. (2014) constructed small-RNA and degradome libraries from wild tomato (*Solanum habrochaites*), and out of these, 192 miRNAs showed increased expression, while expression was decreased in 205 miRNAs. Besides, authors also predicted the miRNA targets and few of the targets were validated using qRT-PCR. Further, most of the target genes were reported to have positive role in chilling response as revealed by their functional analysis via regulating the expression anti-stress proteins and antioxidants (Cao et al., 2014).

### Hypoxia and oxidative stress

Oxygen is extremely important for aerobic organisms including plants. Hypoxia or low-oxygen stress usually results from but not limited to the water logging conditions in the rhizospheric environments of plants. It eventually leads to diminished oxygen availability and thus severely affecting the mitochondrial respiration (Agarwal and Grover, 2006). However, hypoxia also holds significance in signaling. It induces a metabolic shift from aerobic respiration to anaerobic fermentation and significant changes in transcriptome (Moldovan et al., 2010). Recent studies suggested a number of miRNAs as hypoxia-responsive and advocate them to be critical post-transcriptional modulators (Licausi et al., 2011; Zhai et al., 2013). In *Arabidopsis* roots, Moldovan et al. (2010) reported enhanced expression levels of *miR156g, miR157d, miR158a, miR159a, miR172a,b, miR391*, and *miR775* by low oxygen stress.

In *Arabidopsis*, Sunkar et al. (2006) revealed repression of *miR398* and the upregulation of superoxide dismutase (SOD) proteins under oxidative stress. A study of H_2_O_2_-regulated miRNAs from rice seedlings indicated that expression of some miRNAs is regulated in response to oxidative stress; results revealed that out of seven H_2_O_2_-responsive miRNAs i.e., miRNAs that were expressed differentially in H_2_O_2_-treated and control samples; *miR169, miR397, miR827*, and *miR1425* were upregulated while *miR528* was downregulated by H_2_O_2_-treatments (Li et al., 2010).

### Heavy metals (HMs)

Though heavy metals naturally occur in earth's crust, but anthropogenic and industrial activities has led to severe HM pollution in vast areas under cultivation. HM toxicity is one of the major abiotic stresses leading to hazardous effects via altering the physiological and metabolic processes that negatively affects growth, development and productivity of crops (Rascio and Navari-Izzo, 2011; Gupta et al., 2014). HMs can be categorized into two groups, essential and non-essential HMs. Copper (Cu), iron (Fe), zinc (Zn), and manganese (Mn) which at low concentrations plays vital roles in enzymatic and biochemical reactions in plant cell, however are toxic at higher concentrations (Rascio and Navari-Izzo, 2011; Gielen et al., 2012). Whereas non-essential metals consists of cadmium (Cd), aluminum (Al), and mercury (Hg) and are toxic to plants even at low concentrations (Gielen et al., 2012). Though cobalt (Co) was historically not considered as vital for plant growth and development, however, it is now classified as essential micronutrient. Co plays significant roles in growth and development of plant buds, leaf-discs stems and coleoptile, besides assisting in CO_2_ absorption by the plants (Diez, 2014). The HM entry to human food-chain through contaminated crops is a serious human health threat (Gupta et al., 2014). Recent investigations have established miRNA-mediated transcriptional and post-transcriptional regulations of gene expression via base-pairing with their target mRNAs in plants subjected to HM-stress (Yang and Chen, 2013).

#### Cd-toxicity

Cd accumulation by plants results into chlorosis, wilting, growth reduction and cell death, damage to proteins and induces oxidative stress (Zhao et al., 2012). Cd affects crop productivity and its exposure predominantly through food contamination pose serious risks to human health (Rizwan et al., 2016). Steady progress has been made in recent past in investigating the molecular mechanisms involved in Cd toxicity and tolerance/adaptive mechanisms in plants. Aberrant miRNA expression in stressed plant cells and tissues indicated involvement of miRNAs in Cd-responses.

Huang et al. (2010) documented elevated expression of *bna-miR393* in leaves, *bna-miR156a, bna-miR167a/c* in roots and leaves, *bna-miR164b* and *bna-miR394a/b/c* in all tissues of rapeseed (*Brassica napus*) upon Cd exposure with concomitant downregulation of *miR160*. The authors in an attempt to predict the targets generated transgenic *B. napus* lines by overexpressing *miR395* and confirmed its targets to be sulfate transporters (particularly SULTR2;1) and ATP sufurylases (APS) (Huang et al., 2010). SULTR2;1 is vital for its role in sulfate remobilization from mature to younger leaves while APS catalyzes the first step in the sulfur assimilation (Liang et al., 2010). Similarly, Zhou et al. (2012a) reported Cd induced differential expression of numerous conserved and non-conserved miRNAs in *B. napus* roots. Using deep-sequencing analysis, authors tried to understand how Cd regulates genome-wide regulation of miRNA expression and their targets, in all 84 miRNAs including conserved and non-conserved families were identified with most of the miRNAs exhibiting differential expression in shoots/roots under Cd stress (Zhou et al., 2012a). In rice, using microarray assay, Ding et al. (2011) identified 19 Cd-responsive miRNAs from rice seedlings, and their targets were predicted as transcription factors and stress related proteins. In an attempt to decipher the Cd-responsive miRNAs and their mediated gene regulatory networks at the transcriptome level, Xu et al. (2013) constructed small RNA libraries from radish (*Raphanus sativus*) with or without exposed to Cd stress. They identified 15 known and 8 novel miRNA families with notable differential expression in Cd-stressed plants and their targets were then predicted using degradome analysis. Authors postulated the target genes of Cd-responsive miRNAs as phytochelatin-synthase-1, and iron and ABC transporter proteins (Xu et al., 2013).

#### Hg-toxicity

Hg is a highly toxic metal and its ionic form (Hg^2+^) is most prevalent in soil and bioavailable form for plants which are readily taken up through root and aerial part of the plants. It causes toxicity response such as stunted growth, loss of cell shape, vascular abnormality, reduced chlorophyll content and reactive oxygen species (ROS) accumulation (Chen and Yang, 2012). Differential miRNA expressions have been reported as a response to Hg exposure in various plant species, for instance, Zhou et al. (2012b) through deep sequencing approach observed that numerous conserved as well as non-conserved miRNAs were differentially expressed in barrel-clover (*Medicago truncatula*) seedlings subjected to Hg stress. A total of 130 targets for 58 known miRNA families comprising both conserved as well as non-conserved, besides, 37 targets for novel *M. truncatula*-specific candidate miRNAs were identified using degradome sequencing (Zhou et al., 2012b). Authors attributed the differential expression of identified miRNAs and their targets to Hg stress.

An unfortunate consequence of abiotic stresses is the oxidative burst mediated through the excessive ROS generation in plant tissues and cells (Khare et al., 2015). Plants deploy antioxidative armory with enzymatic and non-enzymatic components to detoxify or scavenge these ROS in a systematic way (Khare et al., 2015). Recent reports confirm the role of miRNAs as post-transcriptional regulators of metal induced oxidative stress and responsive antioxidation. It is exemplified by Hg-induced enhanced activity of superoxide dismutase (SOD) in alfalfa (*Medicago sativa*, Zhou et al., 2008). Further, the overexpression of *miR398*-resistant transcript of CSD2 (Cu/Zn-SOD 2) resulted into more CSD2 transcripts and enhanced heavy metal tolerance in transgenic *Arabidopsis* over their non-transformed counterparts (Sunkar et al., 2006), suggesting the apparent involvement of *miR398* in Hg-induced responses.

#### Mn-toxicity

Manganese acts as inorganic catalyst, while Mn and Fe are important components for many enzymatic reactions. Comparably lesser research has been carried out to identify the Mn-toxicity regulated miRNAs, their targets and detailed post-transcriptional regulatory mechanisms in plants in response to Mn stress (Valdes-Lopez et al., 2010; Gupta et al., 2014). Valdes-Lopez et al. (2010) used miRNA macroarray hybridization approach coupled with qRT-PCR to identify Mn-responsive miRNAs in common bean (*Phaselous vulgaris*) and a total of 37 miRNAs displayed differential expression under abiotic stresses including Mn. Out of these, eleven miRNAs were induced whereas another eleven were inhibited under Mn stress. *miR1508, miR1515, miR1510*/2110, and *miR1532* were characterized as Mn-responsive and their targets were predicted as calcium-dependent protein kinase, heat shock proteins, nucleotide-binding site leucine rich repeat resistance like proteins and receptor kinase protein, respectively (Valdes-Lopez et al., 2010).

#### As-toxicity

Arsenic is a class-I carcinogen, widely distributed metalloid present in the earth's crust and enforces severe threats to human health via entering the food chain through contaminated food crops (Srivastava et al., 2011). Investigations have shown that arsenite toxicity in plant tissues leads to reduced photosynthesis rate, perturbed carbohydrate metabolism, besides, generation of ROS, and lipid peroxidation (Jha and Dubey, 2004; Requejo and Tena, 2005). However, little is known about the miRNA-mediated post-transcriptional regulatory mechanisms involved in As responses and tolerance in plants. Therefore, it is interesting to investigate and understand regulatory roles miRNAs play in response to As-stress in plants. In rice, Yu et al. (2012) identified 36 new miRNAs responsive to As and they are involved regulating gene expression in transportation, signaling and metabolism, these finding leads to jasomonic acid (JA) and lipid metabolism in response to As-stress. In addition, Liu and Zhang (2012) reported 67 As-responsive miRNAs from indica rice roots and showed that *miR408, miR528*, and *miR397b* had increased expression, however *miR1316* and *miR390* were repressed under As-stress. Since the role of JA in As stress perception is established (Srivastava et al., 2009), a few miRNAs have been identified to affect JA biosynthesis via targeting the transcription factor involved therein (Gupta et al., 2014), for instance, Srivastava et al. (2012) has reported that *miR319* has been found to be responsive to As stress. This indicates the critical roles of plant miRNAs in regulating JA biosynthesis and which might in turn be involved in metal toxicity responses in plants.

#### Al-toxicity

Al is a major toxicity factor, negatively affecting the crop production on 30–40% of the world's arable land. It binds with COOH and PO_4_ groups present on the root cell wall, which leads to structural changes, inhibition of cell wall expansion and diminished root growth (Ma et al., 2004). This reduces the uptake of minerals nutrient and water. It also affects physiological processes like callose deposition, imbalance cytoplasmic Ca^2+^ and induce oxidative stress (Silva, 2012). In recent years, role of miRNA using high-throughput genome sequencing, Chen et al. (2012) reported altered expression of 23 miRNAs in *M. truncatula* seedlings subjected to Al-stress. Subsequently using same methodology Zeng et al. (2012) identified 30 miRNAs in Al-treated soybean, and interestingly, in this study *miR396* and *miR390* were upregulated unlike earlier observations by Chen et al. (2012), indicating species-specific nature of these miRNAs.

#### Nutrient homeostasis

Mineral nutrients are essential for plant growth, development and crop yield formation. Plants acquire them from their soils and translocate them in cellular compartments. Nutrient homeostasis in plants needs to be maintained for optimal growth and yields. There is increasing evidence to establish critical roles that miRNAs play in nutrient metabolism, besides in maintaining the nutrient homeostasis via regulated-expression of genes involved in uptake and translocation of mineral nutrients in plants (Kehr, 2013; Paul et al., 2015).

Nitrogen (N), Phosphorus (P), potassium (K), and sulfur (S) are vital macronutrients needed for optimal plant growth and development, while various micronutrients such as iron (Fe) and copper (Cu) serve as cofactors of metabolic enzymes and protein complexes in the electron transport chain. Their acquisition, assimilation and metabolism are regulated in plants and deprivation of nutrients affects the production and quality of crop plants. Recently, several novel miRNA were reported for nutrient homeostasis (Fischer et al., 2013; Kehr, 2013; Paul et al., 2015).

Plants respond to phosphate deprivation largely through transcriptional regulation and miRNAs are considered to play pivotal role in phosphate homeostasis (Kant et al., 2011). Several miRNAs have been attributed for the differential regulation of phosphate related gene expression in *Arabidopsis* (Lundmark et al., 2010), rice (Hu et al., 2011), wheat (Zhao et al., 2013), barley (Hackenberg et al., 2013), and maize (Pei et al., 2013). Interestingly, overexpression of osa-*miR827* (which targets SPX-MFS protein family members) resulted into perturbed P homeostasis and mobilization in rice leaves, over-accumulation of Pi in older leaves indicating the regulatory role of miRNA in P metabolism (Wang et al., 2012).

Nitrogen being an essential constituent of nucleic acids, protein and chlorophyll is highly significant for plant growth and survival and its deficiency exerts serious growth retardations (Paul et al., 2015). There are recent reports of miRNA mediated regulation of plant growth and metabolism under N-deficiency. Liang et al. (2012) recorded differential expression of N-deficiency responsive miRNAs in *Arabidopsis*. N-starvation induced the expression levels of *miR160, miR780, miR826, miR842*, and *miR846*, while expressions were repressed in case of *miR169, miR171, miR395, miR397, miR398, miR399, miR408, miR827*, and *miR857*. Interestingly, authors concluded that many of the N-responsive miRNAs were involved in cross-talks between responses to multiple nutrients including N, P, S, and Cu. Authors identified a novel N-deficiency induced *miR826* which targeted AOP gene encoding 2-oxoglutarate-dependent dioxygenase involved in glucosinolate biosynthesis (Liang et al., 2012). Similarly, global miRNA expression profiling was carried out in rice cultivars with contrasting tolerance abilities against N-deprivation (Nischal et al., 2012). Results revealed differential expressions of 32 miRNAs and the predicted target genes included TFs, proteins involved in metabolic processes as well as stress-proteins (Nischal et al., 2012). All these reports establish key roles played by miRNAs in low-N tolerance in crop plants.

Sulfur is essential for plant growth and physiological functioning. Plant roots uptake S in the form of sulfate, which should be reduced to sulfide before it is further metabolized. Sulfur starvation results into physiological imbalances, reduced plant growth, loss of plasticity against environmental stresses and pathogenic attacks, and overall yield reductions. *MiR395* represents a conserved plant miRNA which targets a low-affinity sulfate transporter and ATP sulfurylases (APS) (Jagadeeswaran et al., 2014). S-starvation induced expression of *miR395* is well known in *Arabidopsis* (Kawashima et al., 2009; Jeong et al., 2011; Jagadeeswaran et al., 2014). S-deficiency-specific induction of *miR395* has been reported to be controlled by SLIM1 (SULFUR LIMITATION 1) (Kawashima et al., 2009). The same group reported that *miR395* in association with SLIM fine tune the expression of APS under S-starvation (Kawashima et al., 2011). Jeong et al. (2011) reported that rice plants overexpressing *miR395* displayed S deficiency symptoms. In an interesting study, Jagadeeswaran et al. (2014) reported the upregulation of *miR395* in *Arabidopsis* seedlings in response to arsenate- or Cu^2+^-induced oxidative stress. Authors evidenced that the application of glutathione repressed *miR395* induction in S-starved plants and they concluded the apparent involvement of redox signaling in induced-expression of *miR395* in S-deficient *Arabidopsis* seedlings (Jagadeeswaran et al., 2014).

Copper is essential for plants particularly for photosynthesis. Most common Cu-proteins, the plastocyanin and Cu/Zn-SOD are found in chloroplasts, interestingly though; Cu/Zn-SOD can be replaced by Fe-SOD under limited Cu availability (Abdel-Ghany and Pilon, 2008). Abdel-Ghany and Pilon (2008) reported that *Arabidopsis* plants accumulated *miR397, miR408*, and *miR857* under low Cu availability and authors concluded miRNA-mediated regulation as critical mechanism to regulate non-essential Cu-proteins. The low-Cu induced downregulation of Cu/Zn-SOD involves miRNAs (*miR398*) interferences. *MiR398* is a conserved plant miRNA and as discussed earlier, it target and downregulates CSD1 and CSD2 via inhibiting their mRNA translation. Further, Beauclair et al. (2010) reported that Cu-starvation induces *miR398* also downregulates CCS1 (Cu chaperones for SOD1, which is essential for synthesizing mature Cu/Zn-SOD in *Arabidopsis*). Contrarily, some miRNA families (*miR397, miR408*, and *miR857*) get induced under low amount of Cu and eventually repress the expression of laccase and plastocyanin genes (Paul et al., 2015). In a recent study, Jin et al. (2015) identified several Cu-stress responsive miRNAs in tree peony (*Paeonia ostii*), with 12 conserved and 18 new. The sequence homology search for target prediction revealed multiple targets of many miRNAs, and more than one third targets were involved in biological process (Jin et al., 2015). Authors attributed strong Cu stress tolerance abilities of *P. ostii* to miRNA-mediated gene regulatory mechanisms.

Other minerals, vital for plant growth and development, include Mn, Fe and Zn. Fe and Zn acts as cofactors for numerous key metabolic enzymes (Forieri et al., 2013) and their bioavailability largely depends on their uptake, transport and allocation. Waters et al. (2012) reported downregulation of *miR397a, miR398a*, and *miR398b/c* by Fe-deficiency, suggesting their possible involvement in plant adaptation to Fe stress. Interestingly, authors proposed that the Cu accumulation, miRNA-regulation and concomitant contrasting expression patterns of Fe and Cu-SOD genes are coordinated responses to Fe limitation (Waters et al., 2012). Further, depleted supply of Zn resulted into elevated *miR398* expression coupled with repressed expression of CSD, while downregulation of *miR398* resulted in comparatively steady levels of CSD expression in Sorghum leaves, indicating miRNAs as critical regulators of plant-responses to Zn shortage (Li et al., 2013). In another noteworthy investigation, Marín-González and Suárez-López (2012) demonstrated that miRNAs are involved in nutrient transport and inter-cellular and long-distance signaling in plants.

### UV-radiation

Elevated levels of UV-B radiation as a consequence of ozone-layer depletion negatively affect plant growth and development mainly via ROS-mediated oxidative burst (Kruszka et al., 2012).

In a recent study, Wang et al. (2013) isolated a novel wheat *Tae-miR6000*, and confirmed its expression diversity after UV-B treatments. Additionally, they identified *miR156, miR159, miR164, miR167a, miR171*, and *miR395* as UV-B responsive. Of these, *miR159, miR167a*, and *miR171* were significantly upregulated and the remaining three miRNAs were downregulated, at different time points after UV-B treatment.

## Targeting miRNAs for developing abiotic stress tolerant transgenic plants

Several transgenic strategies are employed in crop improvement investigations with primary objective of improved crop yield and quality (Privalle et al., 2012). The principal concentration of these improvement strategies has been on the abiotic stress and herbicide or pest resistant genes (Kumar et al., 2010; Buiatti et al., 2013). Among all the strategies, miRNA based genetic modification seems most promising since miRNA regulates gene expression at transcriptional or post-transcriptional levels. Several methods can be employed for miRNA manipulations including desired overexpression/repression of stress-responsive miRNAs and/or their target mRNAs, miRNA-resistant target genes, target-mimics and artificial miRNAs (amiRNAs, Zhou and Luo, 2013). Various studies have confirmed the vital roles of miRNAs in multiple developmental as well as signaling pathways in plants. Aberrant expression patterns of these small molecules, which might be stress-, genotype-, tissue-, miRNA-specific, induced by abiotic environment proposes their potential use as target for genetic improvement (Zhang, 2015). Latest advancements in genomics have provided the scientific society with genome wide high throughput sequence data of many important crop species which is a rich source of information about potential candidate transgenes, of which miRNA have become progressively demandable due to their important regulatory role in various phases of plant life cycle (Sun, 2012; Zhou and Luo, 2013; Zhang and Wang, 2016). The list of transgenic plants produced recently via altering the miRNA expression for improving plant tolerance to abiotic stresses is presented in Table 3.

**Table 3:** **Overexpression of single common stress-responsive miRNA for conferring abiotic stress tolerance in model and crop plants**.

**miRNA**	**Source of the targeted miRNA gene**	**Target**	**Transgenic plant**	**Expression strategy**	**Response**	**References**
*miR156*	*Oryza sativa*	SPL	*Oryza sativa*	Overexpression of *OsmiR156k*	Reduced cold tolerance	Cui et al., 2015
*miR172*	*Glycine max*	AP2 like TFs	*Arabidopsis*	Overexpression of *gma-miR172c*	Enhanced Water deficit and salt tolerance	Li et al., 2016
*miR319*	*O. sativa*	PCF5 and PCF8	*Oryza sativa*	RNAi	Enhanced cold tolerance	Yang C. et al., 2013
*miR319*	*O. sativa*	TCP	*Agrostis stolonifera*	Constitutive overexpression of *osa-miR319a*	Enhanced drought and salt tolerance	Zhou et al., 2013
*miR390*	*O. sativa*	SRK	*Oryza sativa*	Overexpression of miR390	Reduced Cd tolerance/enhanced Cd accumulation	Ding et al., 2016
*miR394a*	*G. max*	F-box Protein	*Arabidopsis*	Overexpression of *gma-miR394a*	Enhanced drought tolerance	Ni et al., 2012
*miR394a*	*Arabidopsis thaliana*	LCR	*Arabidopsis*	Overexpression of miR394a/*LCR* loss of function mutant	Enhanced cold tolerance	Song et al., 2016
*miR395*	*A. thaliana*	*BnSultr, BnAPS*	*Brassica napus*	Overexpression of *miR395* driven by CaMV35S promoter	Shorten or no surface trichomes with delayed transition from juvenile to adult vegetative stage	Huang et al., 2010
*miR398*	*A. thaliana*	CSD1, CSD2, CCS	*Arabidopsis*	Loss of function mutants of CSD1 and CCS, knockdown mutant of CSD2	Enhanced thermo tolerance	Guan et al., 2013
*miR399*	*A. thaliana*	IPS-1	*Solanum lycopersicum*	overexpression of *Ath-miR399d* under control of *rd29A* promoter	Better growth performances under phosphorus deficiency and low temperature	Gao et al., 2015
*miR408*	*A. thaliana*	Copper related gene	*Cicer arietinum*	Overexpression of *Athpre-miR408*	Enhanced drought tolerance	Hajyzadeh et al., 2015

*SPL, Squamosa promoter binding protein-like; AP, Apetala; TFs, Transcription factors; TCP, Teosinte Branched Cycloidea and PCF family; PCF, Proliferating cell factors; SRK, Stress responsive leucine rich repeat receptor like kinases; LCR, Leaf curling responsiveness; CSD, Copper/Zinc superoxide dismutase; CCS, Copper Chaperon of CSD; IPS, IFN-β Promoter stimulator; BnSultr, Brassica napus sulfate transporters; BnAPS, B. napus ATP sulfurylases*.

Information regarding RNA mediated stress regulatory networks offers an innovative path for improved stress tolerance on genetic level. Recent reports have revealed that manipulation of miRNA arbitrated gene regulations can help to engineer plants for enhanced abiotic stress tolerance (Zhang and Wang, 2016). Due to their central role in intricate gene regulatory network, miRNAs may prove potent targets for plant improvement, with superior tolerance to abiotic stresses (Zhang and Wang, 2015). Either altered tolerance or sensitivity against various abiotic stresses as equated to their wild types is obtained via miRNA-overexpression in transgenic plants (Table 3). These are some of the promising examples of miRNA grounded manipulations for plant tolerance enhancement with respect to harsh environmental conditions.

The *miR156* is the first documented miRNA in plants, vital for plant development, and works as important signaling molecule for on-time flowering. For moderately longer period it also helps to maintain juvenile state of plants. *MiR156* overexpressing rice plants showed reduced cold tolerance (Cui et al., 2015). The *miR172*, which regulates the expression of AP2-like TFs, is recognized as flowering time controller as well as abiotic stress responder. Overexpression of *gma-miR172* in *Arabidopsis* revealed the enhanced water deficit and salt tolerance (Li et al., 2016). Several studies have suggested that *miR319* is concomitant with multiple abiotic stresses which show upregulation during many of the abiotic stress conditions (Zhou et al., 2010). Overexpression of *osa-miR319a* in creeping bentgrass (*Agrostis stolonifera*) significantly improved the salt and drought tolerance of transgenic plants (Zhou et al., 2013). Transgenic rice overexpressing *miR319* showed enhanced cold tolerance. Recent reports advocated the vital role of *miR390* in plants against abiotic stress (Ding et al., 2016). In rice, *miR390* encodes *OsSRK*, (*O. sativa stress-responsive leucine-rich repeat receptor-like kinase*) which plays crucial role in plant development, resistance against the disease and in response to several biotic and abiotic stress environments. The *miR394* is one of the evolutionary conserved miRNAs which shows aberrant expression pattern under abiotic stress. Circumscribed water loss is observed during leaf transpiration in transgenic *Arabidopsis* plants overexpressing *gma-miR394* which eventually improved the drought tolerance (Ni et al., 2012). On similar basis, Song et al. (2016) demonstrated that transgenic *Arabidopsis* overexpressing *miR394* as well as *LCR* (*LEAF CURLING RESPONSIVENESS*, a target of *miR394*) loss of function *lcr* mutants exhibited enhanced cold stress tolerance, indicating the involvement of *miR394* and its target gene LCR in low-temperature responses of plants (Song et al., 2016).

## Conclusion and perspectives

Since the first report of plant miRNAs in early 2000s, remarkable growth has been witnessed in the studies aimed on plant miRNAs. Ever growing evidence reaffirms that these tiny molecules play pivotal roles in plant growth, development and their responses to environmental cues. In last 10 years, large number of conserved as well as non-conserved stress-responsive plant-miRNAs have been identified from major crop and model plant species and this process is fastened with advent of high throughput or deep sequencing technologies. High throughput sequencing tools have helped researchers for genome-wide miRNA expression profiling under important abiotic stresses. Various computational tools and public web-resources have been developed to facilitate the identification, expression profiling and target predictions of stress-responsive miRNAs, besides archiving them. Technological developments have taken place for effective and quick identification of multiple-miRNAs targets including degradome sequencing. However, it has its limitation in identifying targets for miRNAs, particularly the ones, those target gene expression through translation repression and therefore necessitates better alternatives. However, there is a need for functional characterization of miRNAs as regulators of plant responses to singular as well as multiple abiotic stresses. Similarly, as pointed out in some recent reviews (Zhang, 2015; Zhang and Wang, 2015), the amiRNAs may prove to be important for functional studies.

Owing to the critical roles in post-transcriptional regulation of gene-expression in-response-to abiotic stresses and resultant growth attenuation, miRNAs represents themselves as potent targets to engineer abiotic stress tolerance in major crops through transgenic technologies. Therefore, miRNAs represents potential targets for conferring abiotic stress tolerance in plants through their induced altered expression; however, there is a long way to go for effective use of this strategy for producing abiotic stress tolerant plants.

## Author contributions

VK, VS, TSK, RMD, and SHW drafted the manuscript. VS, VK and TSK prepared the tables and revised the manuscript. All the authors made substantial contribution to the work and approved it for publication.

### Conflict of interest statement

The authors declare that the research was conducted in the absence of any commercial or financial relationships that could be construed as a potential conflict of interest.
