# Supplementary material for: MicroRNAs As Potential Targets for Abiotic Stress Tolerance in Plants
Source: Front Plant Sci. 2016 Jun 14;7:817. doi: 10.3389/fpls.2016.00817 (PMC4906921; doi:10.3389/fpls.2016.00817)

Frontiers in Plant Science
Electronic Supplementary Material
doi: 10.3389/fpls.2016.00817

MicroRNAs as potential targets for abiotic stress tolerance in plants

Varsha Shriram[1], Vinay Kumar[2,]*, R. M. Devarumath[3], Tushar Khare[2] and Shabir H. Wani[4]
[1]Department of Botany, Prof. Ramkrishna More College (Savitribai Phule Pune University), Akurdi, Pune 411044, India
[2]Department of Biotechnology, Modern College (Savitribai Phule Pune University), Ganeshkhind, Pune 411016, India
[3]Molecular Biology and Genetic Engineering Section, Vasantdada Sugar Institute, Pune 412307, India
[4]Division of Genetics and Plant Breeding, Faculty of Agriculture WADURA, Sher-e-Kashmir University of Agricultural Sciences and Technology, Kashmir 191121. India
*Author for correspondence

SUPPLEMENTARY TABLE S1 | Number of publications on miRNA in last 10 years in PubMed

Year	Search Term		
	miRNA	Plant miRNA	Plant miRNA Abiotic Stress
2006	704	138	0
2007	1072	166	6
2008	1741	220	11
2009	2537	242	10
2010	3862	355	25
2011	5145	402	24
2012	6722	512	39
2013	8468	589	46
2014	9893	572	57
2015	10393	573	59

SUPPLEMENTARY Figure S1 | Number of articles on plant miRNAs and abiotic stress specific plant miRNAs in last 10 years in PubMed

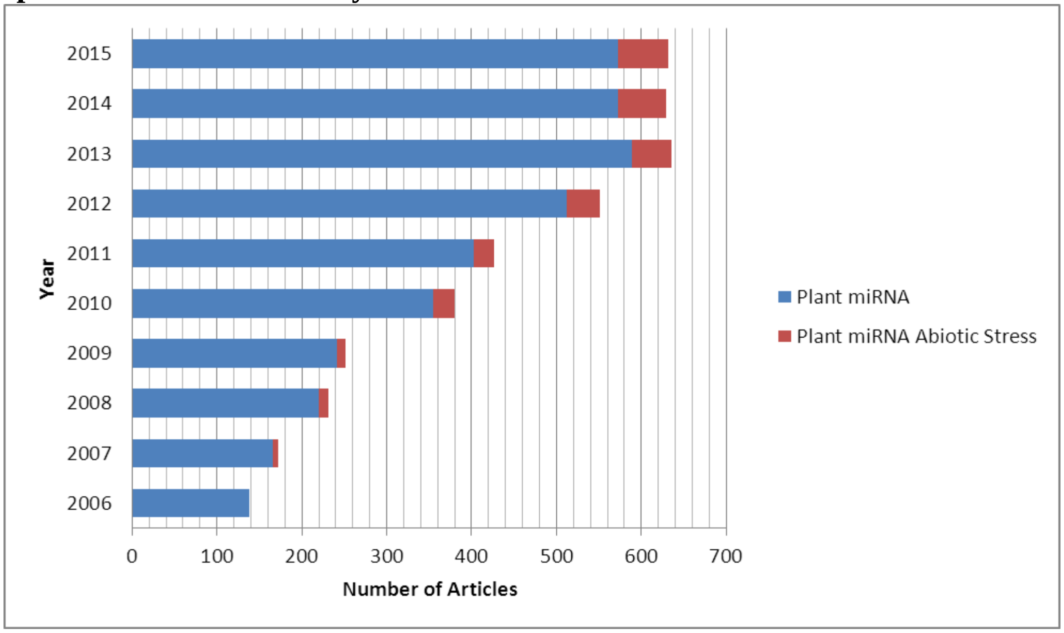

Supplement: Supplementary file 1 [file DataSheet1.pdf]
